# Supplementary material for: Manipulation of the electrical and memory properties of MoS2 field-effect transistors by highly charged ion irradiation
Source: Nanoscale Adv. 2023 Nov 6;5(24):6958–66. doi: 10.1039/d3na00543g (PMC10696994; doi:10.1039/d3na00543g)
Supplement: NA-005-D3NA00543G-s001 [file NA-005-D3NA00543G-s001.pdf]

Electronic Supplementary Material (ESI) for Nanoscale Advances.
This journal is © The Royal Society of Chemistry 2023

Supplementary Information to "Manipulation of the electrical and memory properties of MoS_2 field-effect transistors by highly charged ion irradiation†"

Stephan Sleziona, Aniello Pelella, Enver Faella, Osamah Kharsah, Lucia Skopinski, Andre Maas, Yossarian Liebsch, Antonio Di Bartolomeo and Marika Schleberger*

Faculty of Physics and CENIDE, University of Duisburg-Essen, Duisburg, Germany

**E-mail: marika.schleberger@uni-due.de,*

Phone: +49 (0)203 379-1600

Abstract. Field-effect transistors based on Molybdenum disulfide (MoS_2) exhibit a hysteresis in their transfer characteristics, which can be utilized to realize 2D memory devices. This hysteresis has been attributed to charge trapping because of adsorbates, or defects either in the MoS_2 lattice or in the underlying substrate. We have fabricated MoS_2 field-effect transistors on SiO_2/Si substrates, irradiated these devices with Xe^{30+} ions at a kinetic energy of 180 keV to deliberately introduce defects and studied the resulting changes of their electrical and hysteretic properties. We find clear influences of the irradiation: While the charge carrier mobility decreases linearly with increasing ion fluence (up to only 20 % of its initial value) the conductivity actually increases again after an initial drop of around two orders of magnitude, likely due to the occurence of hopping transport via localized states. We also find a significantly reduced n-doping ($\approx 10^{12}$ cm^{-2}) and a well-developed hysteresis after the irradiation. The hysteresis height increases with increasing ion fluence and enables us to characterize the irradiated MoS_2 field-effect transistor as a memory device with remarkably longer relaxation times ($\approx$ minutes) compared to previous works.

1. SRIM Calculations of Ion-Sample Interaction

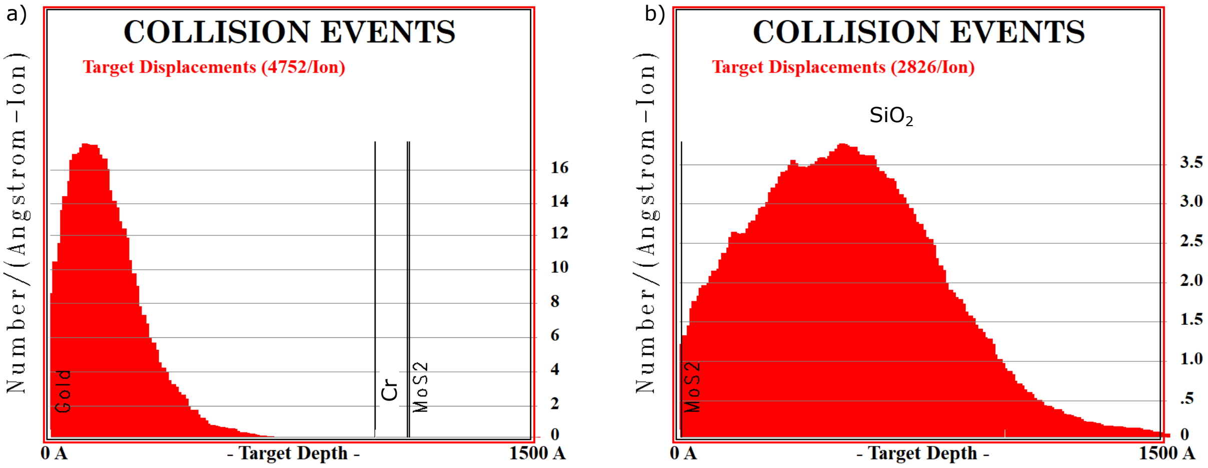

Figure S1. SRIM Calculations for Xe^{+} ions at an kinetic energy of 180 keV where the amount of collision events of the primary ions with the targets atoms is displayed over the targets depth. In a) these collision events are simulated for a system consisting of 100 nm Au and 10 nm Cr layer ontop of a monolayer of MoS2 (0.65 nm thickness). As can be seen, all collisions happen within the Au layer and no collision happen at the Cr/MoS2 interface. In b) these collision events are shown for a target consisting of a monolayer MoS2 ontop of 150 nm layer of SiO_2, representing the conditions for the ions hitting the FETs channel. It is evident that only a fraction of collisions happens in the atomically thin MoS_2 layer and the major amount of collisions happens within the SiO_2.

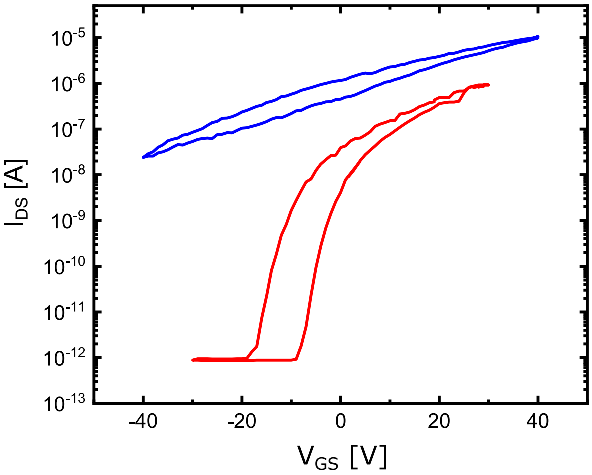

Figure S2. Transfer characteristics of the MoS_2 FET irradiated with a fluence of 100 ions/µm^2 before (blue) and after (red) the irradiation.

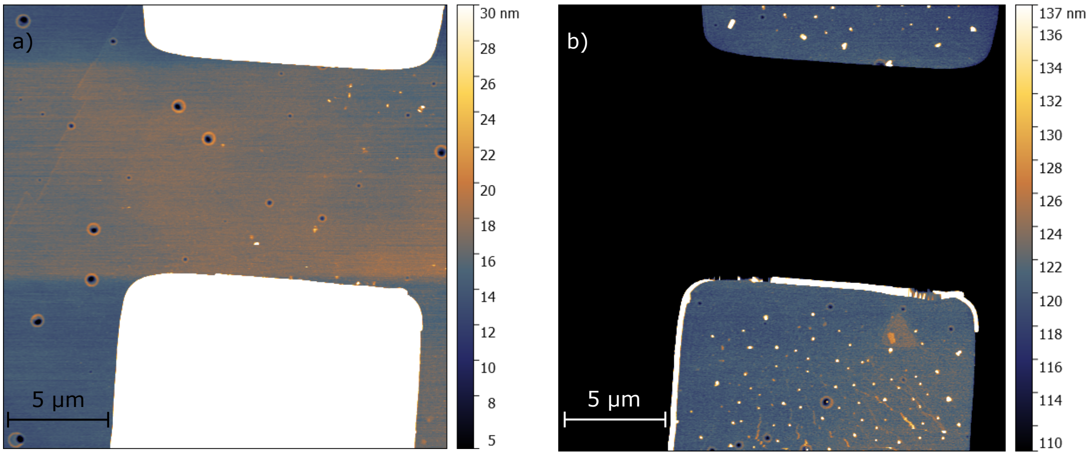

FIGURE S3. 1 AFM topography images of a MoS2-FET after irradiation with Xe^{30+} ions at Ekin = 180 keV with a fluence of 1600 ions/μm^2. a) shows the image with the z-scale adjusted to display surface features on the MoS_2 channel and the substrate, while in b) the z-scale is adjusted to display surface features of the gold contacts. There is no hydrocarbon deposition observable, which is a big advantage for further processing of the device, compared to previous works that used He-ion beams. The mean roughness of the MoS_2 is 0.48 nm and on the gold contacts it is 0.8 nm, which is in the order of magnitude of the noise of the measurement setup. This demonstrates that there is no significant alteration of the topography of our device due to the irradiation. The big protrusions seen distributed randomly over the whole image can happen because of the CVD growth process and are already present prior the ion irradiation
